# Hymenoptera Venom Immunotherapy: Tolerance and Efficacy of an Ultrarush Protocol versus a Rush and a Slow Conventional Protocol

**DOI:** 10.1155/2012/192192

**Published:** 2012-05-24

**Authors:** Vincenzo Patella, Giovanni Florio, Ada Giuliano, Carmine Oricchio, Giuseppe Spadaro, Gianni Marone, Arturo Genovese

**Affiliations:** ^1^Division of Allergy and Clinical Immunology, Department of Medicine, Hospital of Agropoli, ASL, Salerno, 84043 Agropoli, Italy; ^2^Afferent Site to the School Network in Allergy and Clinical Immunology, University of Naples Federico II, Hospital of Agropoli, ASL, Salerno, 84043 Agropoli, Italy; ^3^Laboratory of Enviromental Analysis, Department of Hygiene and Public Health, ASL, Salerno, 84078 Vallo della Lucania, Italy; ^4^Unit of Transfusion Medicine and Immunohematology, Hospital of Agropoli, Salerno, 84043 Agropoli, Italy; ^5^Division of Allergy and Clinical Immunology, Center for Basic and Clinical Immunology Research (CISI), University of Naples Federico II, 80131 Naples, Italy

## Abstract

*Background and Objective*. Various venom immunotherapy (VIT) protocols are available for Hymenoptera allergy. Although adverse reactions (ADRs) to VIT are widely reported, controlled trials are still needed. We conducted a randomized prospective study to evaluate ADRs and the efficacy of three VIT regimens. *Methods*. 76 patients with Hymenoptera allergy, aged 16–76 years, were randomized to receive an ultrarush protocol (group A: 27 patients), a rush protocol (group B: 25), or a slow protocol (group C: 24). Aqueous venom extract was used in incremental phase and an adsorbed depot in maintenance phase. ADRs and accidental Hymenoptera stings during VIT were used to evaluate efficacy. *Results*. During incremental treatment, ADRs occurred in 1.99%, 3.7%, and 3.9% of patients in groups A, B, and C, and in 0.99%, 1.46%, and 2.7%, respectively, during maintenance. ADRs were significantly fewer in group A (incremental + maintenance phase) than in group C (1.29% versus 3.2%; *P* = 0.013). Reactions to accidental Hymenoptera stings did not differ among groups (1.1%, 1.2%, and 1.1%). *Conclusion*. Ultrarush was as effective as the rush and slow protocols and was associated with a low incidence of reactions to stings. This study indicates that ultrarush VIT is a valid therapeutic option for Hymenoptera allergy.

## 1. Introduction

Reactions to stings by Hymenoptera species (*Apis mellifera* and *Vespula Species*) are classified as normal local reactions, large local reactions (LLRs), systemic anaphylactic reactions (SARs), systemic toxic reactions, and unusual reactions [1].All patients who have had an SAR to Hymenoptera stings should avoid insects that sting; they should also carry epinephrine for emergency self-administration, undergo examination for IgE antibodies specific for insect venom, and be considered candidates for venom immunotherapy (VIT) [1, 2]. Immunotherapy with purified Hymenoptera venom reduces the risk of anaphylactic reactions in most patients [2]. The primary goal of VIT is to prevent life-threatening reactions. A secondary benefit is that it helps relieve anxiety about insect stings and improves quality of life [3, 4]. The chance of a subsequent sting causing a more severe reaction than previous sting-induced reactions may be just 1% [5, 6]. Furthermore, a too short interval between stings increases the risk of a systemic reaction [7].

In recent decades, a number of VIT protocols have been proposed with the aim of reducing the number of injections, visits, and risk of SAR [2, 4, 8–10]. Among the various strategies devised to desensitize patients with SAR, an ultrarush protocol has been proposed as an alternative to rush and slow protocols in children, adolescents, and adults [11–18]. One advantage of rapid complete desensitization could be to reduce the risk of relapse of anaphylaxis if the patient is stung before the induction phase is completed. Rush therapy should be indicated for patients with moderate and frequent SAR at a high risk of frequent stings [14] and for patients with a single episode of SAR, but high psychological involvement [1, 13, 19, 20]. Although VIT-associated ADRs have been widely reported, there is a need for controlled, randomized trials that directly compare different regimens, that is, weekly, rush and ultrarush protocols, to determine whether the ultrarush VIT protocol is well tolerated and effective in patients with Hymenoptera sting-induced SAR.

The aim of this study was to evaluate, in a randomized controlled trial, the risk of adverse reactions (ADRs) and the efficacy of an ultrarush protocol compared to rush and weekly regimens in patients allergic to the Hymenoptera species, *Apis mellifera* or *Vespula Species*, commonly known as honey bee (HB) and yellow jacket (YJ).

## 2. Methods

### 2.1. Study Design

Patients with an indication for VIT were treated with commercially available preparations of Hymenoptera venom extracts and monitored for at least 24 months to record side effects and efficacy. According to three different treatment schedules (Table 1), the patients received injections of an aqueous extract of Hymenoptera venom (Aquagen SQ, ALK-Abelló, Hørsholm, Denmark) during the incremental stage of treatment and a purified aluminium-adsorbed depot extract of Hymenoptera venom (Alutard SQ, ALK-Abelló) during the maintenance stage. Both preparations were certified and prepared from the same source by the supplier (ALK-Abelló). The venom was purified by a Sephadex-gel filtration process by which the protein fractions are separated according to molecular weight. Venom extracts do not contain vasoactive amines. In addition, the extract undergoes filtration to reduce the presence of small peptides like apamin, kinins, and mast cell degranulating peptides. For depot VIT, the raw venom undergoes the same purification procedure that results in the recovery of a fraction containing only allergen, which is subsequently adsorbed onto aluminum hydroxide. All preparations contain the same amount of allergens, that is, 100 *μ*g/mL, as specified by the manufacturer (ALK-Abelló). This ensured homogeneous immunogenicity. Injections were carried out according to EAACI Position Papers [2, 21].

### 2.2. Cohort and Randomization of Patients

Seventy six patients (51 males, 25 females; aged 16–76 years) with history of SAR to HB or YJ venom were randomly assigned to different treatment for VIT as reported in Table 2. Patients were randomly assigned to group A, B, or C using a computer-generated random list and numbered envelopes. The envelopes were opened immediately before the start of venom immunotherapy. Patients of groups A (*n* = 27), B (*n* = 25), and C (*n* = 24) were treated with an ultrarush, rush, and weekly protocol, respectively. Groups A and B were admitted to hospital for VIT injections. Adverse reactions were graded according to Müller's classification [1, 9].

### 2.3. Incremental and Maintenance Phases

Group A patients were treated with a three-hour ultrarush protocol (Table 1a) [22]. Patients underwent continuous measurements of oxygen saturation and repeated measurements of blood pressure. The cumulative dose of Hymenoptera venom (HB or YJ) at the end of the incremental phase was 111.101 *μ*g. Thirty minutes after the last injection, patients were discharged to home. Subsequently, on day 15, and once a month thereafter, the patients of each group received a subcutaneous injection of 50 *μ*g adsorbed preparation on each arm, according to the parameter reported by the manufacturer (ALK Abelló, Milan, Italy): 100 *μ*g of aqueous preparation = 100 000 SQ units of aluminum-adsorbed depot preparation. Group B patients received the aqueous preparation according to a daily rush schedule as reported in Table 1 (Group B) [23]. Group C was treated weekly with the slow conventional protocol shown in Table 1 (Group C) [22]. The cumulative doses of venom extract during the incremental phase in each group exceeded 100 mcg. No patient enrolled in this study received premedication before VIT.

### 2.4. Skin Prick Test

A skin test was done in all patients at least three weeks after the last sting [1, 24–26]. Sensitization was detected by skin prick tests with concentrations of 1, 10, and 100 *μ*g/mL of Hymenoptera venom. If the prick test was negative, an intradermal test was done with 0.02 mL of venom concentrations from 0.001 to 1 *μ*g/mL, injected into the volar surface of the forearm. The concentration was increased in 10-fold increments until there was a positive response or up to a maximum concentration of 1 *μ*g/mL. Skin response was assessed after approximately 15–20 minutes. Physiological saline and histamine dihydrochloride 0.1% served as negative and positive controls, respectively.

### 2.5. IgE Assay

Total and specific IgE were measured in the patients' serum at diagnosis and 6, 12, 18, and 24 months after reaching the maintenance dose, using the Immulite 2000 Allergy system, according to the manufacturer's instructions (Diagnostic Products Corporation). The linear range of the assay was 0.2–100 kU/L for the Immulite 2000 sIgE method [27, 28].

### 2.6. Evaluation of Tolerance and Efficacy

Tolerance to VIT was evaluated on the basis of the ADRs recorded during the immunotherapy. The adverse reactions were classified as a large local reaction when there was swelling with a diameter more than 10 cm at the injection site that lasted for more than 24 hours (i.e., an LLR), or as a systemic anaphylactic reaction of different grades, as reported in Müller's classification [9]. The efficacy of VIT was evaluated on the basis of the outcome when a patient was accidentally restung by a Hymenoptera species. Patients were asked to report any of such stings during and after the VIT, and the type of reaction, according to Müller's classification [9]. Each patient had followup visits at the start of VIT and at 6, 12, 18, and 24 months thereafter from start of maintenance phase.

### 2.7. Statistical Analysis

Results are expressed as means ± SEM. Treatment groups (A, B, and C) and shift of preparations (from aqueous to depot) were compared. Categorical variables were compared by the chi-square test. Trends within a patient group were quantified by the Wilcoxon signed rank test. When the data were subjected to linear correlation analysis, correlations were calculated using the Spearman rank coefficient (rs) [29]. The level of statistical significance was *P* < 0.05.

## 3. Results

### 3.1. Tolerance of Each Venom Immunotherapy Regimen

**Figure 1:**
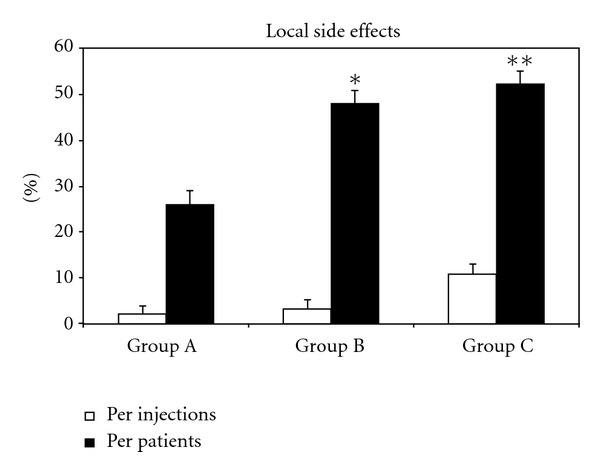
Frequency of local side effects in three groups of patients treated according to different venom immunotherapy (VIT) protocols (see Methods for treatments). Results are shown per injections (□) and per patients (▪). Each bar represents the mean ± SEM. *P < 0.05 compared with the corresponding group A versus group B. **P < 0.001 compared with the corresponding group A versus group C.

**Figure 2:**
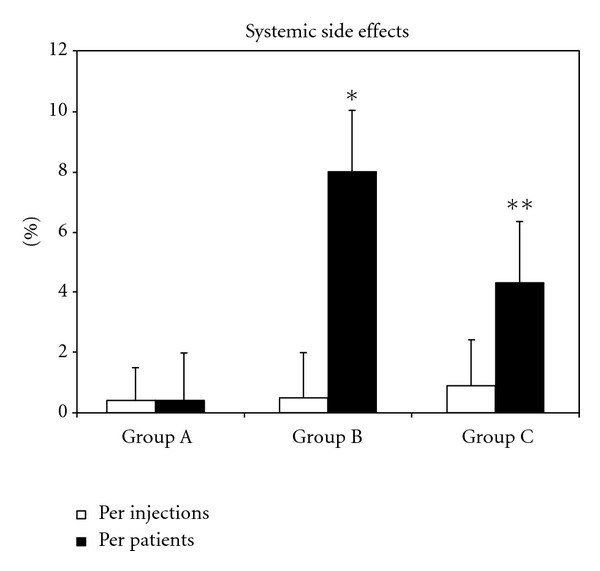
Frequency of systemic side effects in three groups of patients treated according to different venom immunotherapy (VIT) protocols. Results are shown per injections (□) or per patients (▪). Each bar represents the mean ± SEM. *P < 0.001 compared with the corresponding group A versus group B. **P < 0.05 compared with the corresponding group A versus group C.

During the incremental phase of venom immunotherapy, the numbers of LLR (defined as a swelling of more than 10 cm in diameter, lasting longer than 24 h) [9] were 7 out of 351 injections in group A (1.99%); 12/375 in group B (3.2%); 13/356 in group C (3.6%) (Figure 1). During the incremental phase, no SAR occurred in patients in group A (0/351 injections); two SARs occurred in group B (2/375; 0.9% of injections); one in group C (1/356; 0.56% of injections) (Figure 2). The rate of ADRs did not differ among the three groups: group A 7/351 (1.99%); group B 14/375 (3.7%); group C 14/356 (3.9%); (*P* = 0.27). During the incremental phase, two patients of group C withdrew from the study (at the fourth and seventh weeks). Adverse reactions were not the reasons for withdrawal. No patients in groups A and B withdrew from the trial.

In agreement with previous reports [30, 31], SARs occurred more frequently during the maintenance phase in patients with the HB venom preparation than in those receiving the YJ preparation (Table 3). In the maintenance phase, the ADRs were as follows: group A 8/810 (ADR/injections) (0.99%); group B 11/750 (1.46%); group C 12/440 (2.7%); *P* = 0.035; A versus C, *P* = 0.02; B versus C, (*P* = 0.03).

During the whole study (incremental phase + maintenance phase), the ultrarush protocol (group A) resulted in less ADRs than the slow protocol (group C) (1.29% versus 3.2%; (*P* = 0.013)).

### 3.2. Shifting from the Incremental to the Maintenance Phase

No severe ADRs were observed when patients in groups A, B, and C were shifted from the aqueous preparations used during the incremental phase to adsorbed preparations for maintenance, which is in accordance with a previous report [32]. Rates of reactions were similar in each group: group A 1/27 (gastrointestinal symptoms 1), group B 2/25 (LLR 1; gastrointestinal symptoms 1), and group C 1/24 (headache 1).

### 3.3. Total and Specific IgE Levels and Evaluation of Serum s-IgE/Total IgE Ratio with SAR

**Figure 3:**
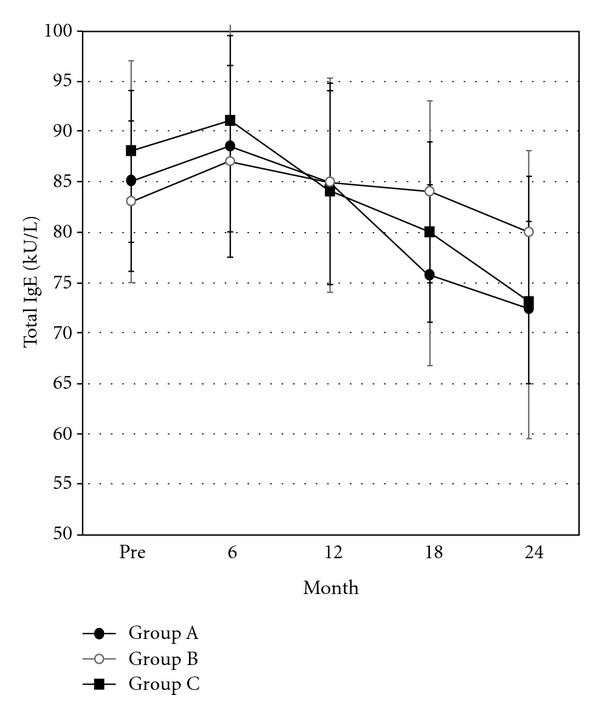
Total IgE during the incremental and maintenance phases in group A (Vespidae 18; Apidae 9; *n* = 27), group B (Vespidae 16; Apidae 9; *n* = 25), and group C (Vespidae 16; Apidae 8; *n* = 24). Vertical bars indicate the mean ± SEM.

**Figure 4:**
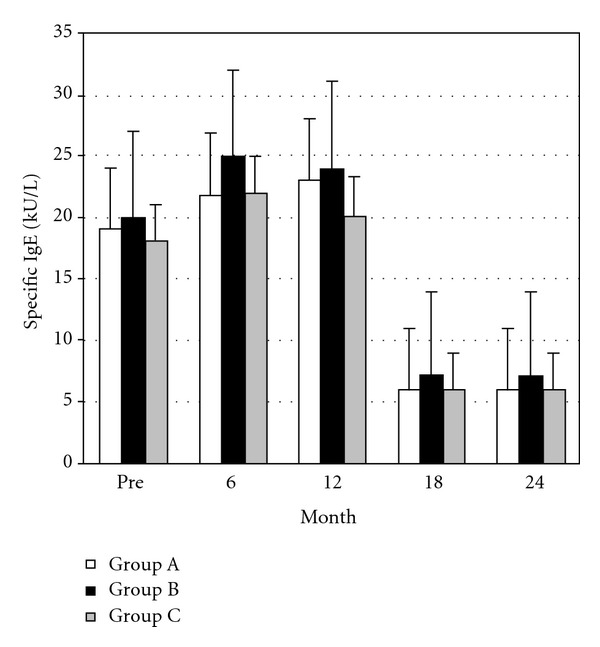
Specific IgE during the incremental and maintenance phases in group A (□) (Vespidae 18; Apidae: 9; *n* = 27), group B (▪) (Vespidae 16; Apidae 9 *n* = 25), and group C (▪) (Vespidae 16; Apidae 8; *n* = 24). Bars indicate the mean ± SEM.

Total and specific IgE was monitored before starting VIT, at the end of VIT, and during the study in the three groups (Figures 3 and 4). The serum s-IgE/total IgE ratio has been reported to predict the clinical response to allergen-specific immunotherapy [33]. We compared the serum s-IgE/total IgE ratio in our three groups of patients using the number of SARs before VIT and after hymenoptera sting during the maintenance phase. There was a similar highly significant direct correlation between the sIgE/tIgE post/preultrarush VIT delta and the SAR pre/postultrarush VIT delta in the three groups considered: group A (rho = 0.79; *P* = 0.034, Spearman rank correlation test), group B (rho = 0.83; *P* = 0.039, Spearman rank correlation test), and group C (rho = 0.77; *P* = 0.041, Spearman rank correlation test).

### 3.4. Efficacy of Venom Immunotherapy

 Accidental Hymenoptera stings during VIT were used to evaluate the efficacy of each of the three desensitization protocols. Of the 76 patients in the maintenance phase, 34 were restung accidentally, no allergic reactions were reported in 23 patients of these 34 patients (group A: 8; group B: 7; group C: 8) (Table 4). In the remaining 11 patients, there were three episodes of LLR and one of SAR (group A), three episodes of LLR and one of SAR (group B), and C two episodes of LLR and one of SAR (group C) (Table 4).

## 4. Discussion

Various protocols have been proposed to obtain rapid desensitization of patients allergic to Hymenoptera venom [11, 12], but how to increase rapidly the doses is still debated. Some studies reported a high risk of ADR with rush protocols [31, 34, 35], while other studies showed that they were safe [13–15, 36–40]. In contrast with a previous study in which systemic reactions were found during the incremental phase of an ultrarush VIT protocol [12], no systemic reaction occurred in our group A patients, whereas they occurred in both groups B and C patients. However, in the maintenance phase, there was a case of SAR in group A, 5 in group B, and 2 in group C. We are unable to explain these findings; notwithstanding, no pretreatment, in terms of antihistamines and corticosteroids, was administered in our patients, unlike several previous studies [40, 41].

A wide range of systemic reactions have been reported during the incremental phase of VIT. Birnbaum et al. used an ultrarush VIT protocol similar to ours to treat 258 Hymenoptera venom-allergic patients with a cumulative dose of 101.1 *μ*g, administered over a period of 3.5 hours. In 325 ultrarush immunotherapies performed, 33 (12.79%) patients experienced a systemic reaction during dose increment, namely, localized urticaria and/or angioedema and/or erythema in 24 patients and hypotension in 9 patients [14]. Bernstein et al. reported mild systemic reactions in only 5.2% of 77 patients; however, all patients received a cumulative total dose of only 58.55 *μ*g on one day followed by an accelerated build-up over three weeks [38]. In our study, the frequency of reactions was comparable with the low number of ADRs observed in previous study, where the side effects during VIT were determined by percent of injections during the incremental and the maintenance phases [34, 42].

In our study, during the maintenance phase, in agreement with previous reports [30], ADRs occurred more frequently in our patients treated with the HB venom preparation than in those receiving the YJ preparation. We previously found that ultrarush VIT rapidly decreased ICAM-1 levels in patients with Hymenoptera allergy [43]. It is likely that the known ability of VIT to correct the imbalance in T lymphocyte subpopulations and in the associated production of cytokines may account for the different response to HB versus YJ venom [43]. In fact, these cytokines include IL-4 and TNF-alpha, which upregulate adhesion molecules [44]. In particular, a shift in cytokine responses from a Th2 to a Th1 pattern was demonstrated during rush VIT using both HB [45] and YJ venom [46]. Regarding T-reg, a recent study found an elevated IL-10 production by CD3(+) T cells few hours after rush VIT [47]. In our study, patients were treated with aqueous extracts of Hymenoptera venom during the incremental phase and well tolerated the shift to the maintenance dose with aluminum hydroxide-adsorbed extracts, confirming previous data [32, 48]. During VIT, the incidence of ADRs due to restings was similar in all three groups. The efficacy of ultrarush therapy is therefore comparable with that of rush and slow conventional protocols. After the incremental phase and also during maintenance treatment, specific IgE levels changed to the same extent in the three treatment groups, in agreement with other studies [49, 50]. The ultrarush protocol significantly reduced total and specific IgE levels as the rush and conventional protocol does. In all patients, there was a highly significant direct correlation between the sIgE/tIgE post/preultrarush VIT delta and the SAR pre/postultrarush VIT delta, which confirms the effectiveness of the three protocols.

Given our observation that the ultrarush VIT protocol is well tolerated and effective together with the fact that patients could be vaccinated before the Hymenoptera season starts [51], the fast protocol (3 hours) means patients have more time available for social activities and work. The working environment of some patients (beekeepers, farmers, etc.) with Hymenoptera allergy is an adjunctive risk factor for insect sting compared with the general population. Effective VIT frees these patients and their families from the worry of stings. Because of its short duration, ultrarush VIT is more easily accepted by the patients and has the additional advantages of rapid protection with a low cost. VIT improves the quality of life in all patients allergic to Hymenoptera venom, particularly those in the Müller classes III and IV and in the people who have been restung during VIT [3, 19, 52].

In conclusion, ultrarush was as effective as the rush and slow conventional protocols and was associated with a low incidence of reactions to Hymenoptera stings. This study indicates that ultrarush VIT is a valid therapeutic option for patients with Hymenoptera allergy.

## Figures and Tables

**Table 1:** Protocol of incremental treatment for ultrarush*, rush^§^, and slow conventional therapy*.

Group A			Group B				Group C		
*μ*g/dose	Cumulative *μ*g/dose	Minute	*μ*g/dose	Cumulative *μ*g/dose	Day	Hour	*μ*g/dose	Cumulative *μ*g/dose	Week
0.001	0.001	0	0.01	0.01	1	0	0.02	0.02	1
0.01	0.011	15	0.1	0.11		2	0.04	0.06	2
0.04	0.051	30	1	1.11		4	0.08	0.14	3
0.05	0.11	45	2	3.11		6	0.2	0.34	4
0.1	0.21	60	3	5.11	2	0	0.4	0.74	5
0.4	0.61	75	3.5	9.61		2	0.8	1.54	6
0.5	1.11	90	3.5	13.11		4	2	3.54	7
1	2.11	105	10	23.11	3	0	4	7.54	8
4	6.11	120	15	38.11		2	8	15.54	9
5	11.11	135	15	53.11		4	10	25.54	10
10	21.11	150	20	73.11	4	0	20	45.54	11
40	61.11	165	25	98.11		2	40	85.54	12
50	111.101	180	25	123.11		4	60	145.54	13
			30	153.11	5	0	80	225.54	14
			35	188.11		2	100	325.54	15
			35	223.11		4			

*As reported in Patella et al. [22].

^§^As reported in Bilò et al. [23].

**Table 2:** Demographic and clinical data of the 76 patients enrolled in the study.

Group	N =	Treatment	Vespula/Apis	Sex (M/F)	Age (range)	Age (mean)	Local large reactions*	Systemic allergic reactions^§ ^	Grade^§ ^ (I) (II)(III)(IV)
A	27	Ultrarush	18/9	19/8	16–76	39.1	1	26	(3) (3) (12) (8)
B	25	Rush	16/9	16/9	18–68	40.3	1	24	(5) (4) (13) (2)
C	24	Slow Conventional	16/8	16/8	19–69	38.6	2	22	(2) (6) (10) (4)

Total	76	—	50/26	51/25	16–76	39.3	4	72	(10)(13)(35)(14)

*It is defined as a swelling exceeding a diameter of 10 cm which lasts longer than 24 h.

^§^Classified according to Müller [9]: grade I: urticaria, pruritus, and malaise; grade II: angioedema, chest tightness, nausea, vomiting, abdominal pain, and dizziness; grade III: dyspnoea, wheeze, stridor, dysphagia, and hoarseness; grade IV: hypotension, collapse, loss of consciousness, incontinence, and cyanosis.

**Table 3:** Side effects of patients treated with maintenance dose.

	Group A	Group B	Group C	Total
Patients	1	7	6	14
maintenance dose of *Apis m. *	1	6	5	12
maintenance dose of *Vespula spp. *		1	1	2
Local large reaction* (%)	—	2(33.3)	4(66.6)	6
Systemic anaphylactic reaction^§^ (%)	1(12.5)	5(62.5)	2(25.0)	8
Grade I	1	4	1	6
Grade II	—	1	1	2

*A large local reaction is defined as a swelling at the site of more than 10 cm lasting for more than 24 hours [31].

^§^A systemic anaphylactic reaction (SAR) was classified with modified classification of Müller [9].

**Table 4:** Allergic reactions to a field sting.

	Group A	Group B	Group C	Total
Patients restung	13	10	11	34
maintenance dose of *Apis m. *	9	6	7	22
maintenance dose of *Vespula spp. *	4	4	4	12
No local and systemic effects	8	7	8	23
Local large reaction*	3	3	2	8
Systemic anaphylactic reaction^§^	1	1	1	3
Grade I	—	1		1
Grade II	1	—	1	2
Grade III	—	—	—	—
Grade IV	—	—	—	—

*A large local reaction is defined as a swelling at the site of more than 10 cm lasting for more than 24 hours.

^§^A systemic anaphylactic reaction (SAR) was classified with modified classification of Müller [9].

## Abbreviations

ADR: Adverse reaction
HVA: Hymenoptera venom allergy
LLR: Large local reaction
SAR: Systemic anaphylactic reaction
VIT: Venom immunotherapy.
